# Resistance Mutations A30K and Y93N Associated with Treatment Failure with Sofosbuvir and Daclatasvir for Hepatitis C Virus Infection Non-Responder Patients: Case Reports

**DOI:** 10.3390/v11111004

**Published:** 2019-10-31

**Authors:** Vanessa D. Costa, Patricia Pellegrini, Vivian Rotman, Ana Maria Pittella, Estevão P. Nunes, Barbara V. Lago, Elisabeth Lampe, Francisco C. A. Mello

**Affiliations:** 1 Laboratório de Hepatites Virais, Instituto Oswaldo Cruz, FIOCRUZ, Avenida Brasil, 4365–Manguinhos, 21040-900 Rio de Janeiro, RJ, Brazil; barbaravlago@gmail.com (B.V.L.); elisabeth.fiocruz@gmail.com (E.L.); 2 Serviço de Hepatologia, Universidade Federal do Rio de Janeiro, Rua Professor Paulo Rodolpho Rocco, 255, Cidade Universitária, 21044-020 Rio de Janeiro, RJ, Brazil; prpellegrini@terra.com.br (P.P.); vrotman@gmail.com (V.R.); 3 Hospital Quinta D’Or. Rua Almirante Baltazar, 435, São Cristóvão, 20941-150 Rio de Janeiro, RJ, Brazil; florcatita@gmail.com; 4 Instituto Nacional de Infectologia Evandro Chagas, INI/FIOCRUZ, Avenida Brasil, 4365-Manguinhos, 21040-360 Rio de Janeiro, RJ, Brazil; estevao.portela@gmail.com

**Keywords:** hepatitis C virus (HCV), DAA, resistance

## Abstract

In Brazil, hepatitis C treatment has been evolving significantly with the licensing of direct-acting antivirals (DAAs). However, viral determinants (amino acid substitutions in hepatitis C virus (HCV) genome and infective genotype) associated with host factors (hepatic condition and prior HCV therapy) might limit the achievement of sustained virologic response (SVR). Here, we described two case reports in which the occurrence of HCV NS5A mutations A30K (subtype 3a) and Y93N (subtype 1a) might have influenced daclatasvir (DCV)/sofosbuvir (SOF) combined therapy non-response. Despite high response rates for DAA combined therapies in Brazil, these case reports stated the importance of an investigation about how to manage a DAA treatment failure since a combination of factors, especially the occurrence of resistance substitutions, could impact a rescue therapy with new available antivirals in clinical routine.

## 1. Introduction

It is estimated that 71 million people worldwide are chronically infected with hepatitis C virus (HCV) [1]. Genotype distribution in Brazil indicated a prevalence of HCV subtypes 1a, 1b and 3a [2]. In 2015, oral combinations of direct-acting antivirals (DAAs) targeting HCV non-structural proteins NS5A and NS5B were included in Clinical Guidelines for the Treatment of Hepatitis C and Coinfections [3] published by Brazilian Ministry of Healthy. Daclatasvir (DCV), a NS5A inhibitor, in combination with sofosbuvir (SOF), a nucleotide analogue inhibitor of NS5B, is a daily regimen administrated with or without ribavirin for Brazilian patients infected with HCV genotypes 1 and 3. Despite its high rate of effectiveness in viral clearance, host and virus factors associated with treatment failure, which include liver fibrosis, negative response to previous therapy and resistance associated-substitutions (RASs), could limit the effectiveness of these drugs in achieving sustained virological response (SVR) [4]. NS5A amino acid substitutions A30K (subtype 3a) and Y93N (subtype 1a) were described in literature in vitro [5,6] and in vivo [7,8,9,10] as responsible for reducing DCV action. Regarding NS5B gene, previous studies reported that substitutions on residues S282 and V321 can reduce susceptibility to SOF [11,12]. We report here two cases of chronic hepatitis C patients infected with HCV subtypes 1a and 3a who failed DCV/SOF combined therapeutic regimen. Treatment failure might have been related to the occurrence of NS5A RASs Y93N and A30K which were identified in persistent HCV strains recovered after the end of unsuccessful treatment.

## 2. Materials and Methods

Two patients who failed to achieve viral clearance 12 weeks after the end of DCV/SOF combined therapy were attended at Ambulatory of Viral Hepatitis/FIOCRUZ, Rio de Janeiro. Serum sample from patient 1 and patient 2 were collected in October 2016 and November 2017, respectively. A written informed consent was obtained from patients as established in the ethics statement approved by the ethics committee from Oswaldo Cruz Foundation (CAAE 68116417.2.0000.5248). Molecular tests included HCV RNA extraction using commercial reagent High Pure Viral Nucleic Acid Kit (Roche Life Science, Mannheim, Germany) following manufacturer’s recommendations. After, HCV NS5A and NS5B genes were amplified by one-step reverse-transcription (RT) with polymerase chain reaction (PCR) followed by a second round of PCR (nested-PCR) through reagents from Superscript III One Step RT-PCR system (Thermo Fisher Scientific, Waltham, MA, USA) and Platinum *Taq* DNA Polymerase High Fidelity (Thermo Fisher Scientific). Specific primers used for each subtype and PCR conditions were described in previous study by Costa et al. (2019) [10]. Amplified products were submitted to purification using High Pure PCR Product Purification Kit (Roche Life Science) and double-stranded DNA concentration was estimated by Qubit dsDNA BR Assay Kit (Thermo Fisher Scientific). Additionally, nucleotide sequencing was performed with Big Dye Terminator v3.1 Cycle Sequencing kit (Applied Biosystems, Foster City, CA, USA) according to the manufacturer’s instructions and analyzed on ABI 3730 DNA automated sequencer (Applied Biosystems). Reference HCV sequences from subtypes 1a and 3a were obtained from the Los Alamos HCV Sequence Database [13]. Nucleotide sequences retrieved from both strands were assembled to generate a consensus sequence and a multiple sequence alignment was analyzed in MEGA version 7.0 [14]. Deduced amino acid composition of NS5A and NS5B proteins was evaluated for the presence of RASs at residues previously reported as associated to drug resistance (NS5A: M28, Q/A30, L31 and Y93; NS5B: L159, S282 and V321) [15].

## 3. Results

### 3.1. Case Report 1

**Figure 1:**
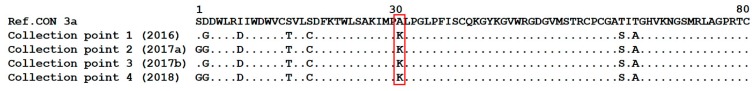
NS5A deduced amino acid sequence alignment comparing a consensus hepatitis C virus (HCV) subtype 3a sequence and four post-treatment collection points of patient 1. Substitution in residue 30 (A30K) is indicated in a red box.

Patient 1 was a cirrhotic 65-year-old woman from Northeast Brazil (Alagoas State). The anamnesis revealed that patient 1 was diagnosed with chronic hepatitis C (HCV subtype 3a) in 2011. In 2013, patient 1 was submitted to antiviral therapy with pegylated interferon and ribavirin. Treatment was discontinued due to occurrence of thrombocytopenia. In 2016, a retreatment with DCV/SOF and ribavirin for 12 weeks was conducted in a private hospital in Rio de Janeiro. Patient adherence to treatment was satisfactory as she concluded the 12-week period of therapy. Molecular diagnosis by real-time PCR indicated that post-treatment HCV viral load was 9360 IU/mL (3.97 Log IU/mL). Laboratory biochemical tests showed ALT of 18 IU/L, AST of 49 IU/L and gamma glutamyl transpeptidase (GGT) of 134 IU/L. In order to investigate viral molecular factors that could have influenced non-response status, resistance analyses were requested by physicians after referring patient 1 to our Ambulatory of Viral Hepatitis in FIOCRUZ. The presence of HCV RNA was confirmed by qualitative NS5A gene RT-PCR amplification and posterior nucleotide sequencing [10]. Sequence assembly and deduced amino acid residues alignment with reference HCV subtype 3a sequence (Ref.CON_3a) identified the presence of RAS A30K. No RASs in NS5B deduced amino acid residues were observed. Besides resistance analysis after 12-week therapy with DCV/SOF in 2016, patient 1 had two other serum samples collected in 2017 and 2018. Nucleotide sequencing results from both samples demonstrated the persistence of RAS A30K in NS5A protein two years after the end of treatment (Figure 1).

### 3.2. Case Report 2

Patient 2 was a 67-year-old Brazilian male who had been diagnosed with HCV genotype 1a infection in 2011. In anamnesis, patient reported type 2 mellitus diabetes (MD). Glibenclamide and metformin was administrated for treating MD. In June 2012, a liver biopsy revealed advanced fibrosis (METAVIR score F3). Serological and biochemical tests performed in May 2013 showed that patient 2 was anti-HBc positive, HBsAg and anti-HBs negative (serological evidence of past hepatitis B infection), alanine aminotransferase (ALT) of 64 IU/L and aspartate aminotransferase (AST) of 59 IU/L. In December 2013, patient 2, until then therapy-naive, started antiviral treatment with DAA telaprevir 375 mg (3 tablets every 12 h) plus peginterferon alfa-2a (180 μg/week) and ribavirin (250 mg). HCV RNA viral load before this triple therapy was 1,820,817 IU/mL (6.26 Log IU/mL). Treatment was well-tolerated in the first three weeks; however, at week 4, patient 2 had episodes of myalgia and asthenia. HCV RNA viral load evaluated at week 12 and week 16 was 446 IU/mL (2.65 Log IU/mL) and 95,904 IU/mL (log 4.98 Log IU/mL), respectively. Due to the increase in HCV viral load, therapy was suspended and patient 2 was considered non-responder (absence of 2 log decline in HCV RNA levels between weeks 12 and 16). Laboratory biochemical tests performed after the treatment cessation showed ALT levels of 70 IU/L and AST levels of 45 IU/L. 

**Figure 2:**
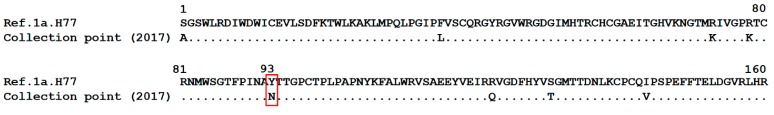
NS5A deduced amino acid sequence alignment comparing a consensus HCV subtype 1a sequence and a post-treatment collection point of patient 2. Substitution in residue 93 (Y93N) is indicated in a red box.

In May 2016, after the inclusion and availability of new DAAs in Brazil, physicians prescribed a retreatment for patient 2. In October 2016, patient 2 began a combined 24-week interferon-free therapy with DCV (60 mg/daily), SOF (400 mg/daily) and ribavirin (5 tablets daily). After 24 weeks of combined therapy, HCV viral load was undetectable; however, SVR 12 weeks after the end of treatment was not achieved, configuring a virologic relapse. In order to verify the occurrence of drug resistance profile in ongoing HCV infection, physicians had requested molecular tests in a serum sample collected seven months after the end of treatment. After nucleotide sequencing of HCV strain infecting patient 2, deduced NS5A amino acid sequence was aligned against HCV genotype 1a reference sequence H77 and primary RAS Y93N was identified (Figure 2). No RASs in NS5B amino acid sequence were observed. In spite of treatment failure with viral persistence, overall clinical condition of patient 2 was considered healthy by physicians after therapeutic regimen with DCV/SOF. Based on this, medical staff decided that patient 2 should wait for the upcoming DAAs before attempting a retreatment to clear HCV viral infection.

## 4. Discussion

In Brazil, routine clinical practices in hepatitis C treatment had reported SVR rates higher than 95% after the availability of interferon-free combined DAAs therapies [10,16]. Despite the high efficacy rate of new DAAs, previous studies had pointed out that a combination of viral factors such as HCV infecting genotype and drug-specific mutations in HCV genome could have major influence in therapeutic response. According to Buti et al. (2016) [17], infection with HCV subtypes 1a and 3a strains and the presence of NS5A RASs in specific amino acid residues are associated with reduction in DAAs susceptibility. Based on data from previous clinical trials from Nelson et al. (2015) [18] and Poordad et al. (2016) [19], presence of baseline NS5A RASs in viral subpopulations seems to reduce DCV effectiveness in patients with advanced cirrhosis.

Regarding case report 1, negative predictive factors such as HCV subtype 3 infection and cirrhotic condition could have influenced an unsatisfactory therapy outcome. Quantitative molecular analysis and biochemical tests at the end of DCV/SOF treatment indicated detectable HCV viral load (3.97 Log UI/mL) with elevated hepatic enzymes AST and GGT. In addition to HCV infecting genotype and hepatic condition, resistance analyses demonstrated the presence of amino acid substitution A30K in NS5A protein. Hernandez et al. (2013) [7] reported that viral strains with amino acid substitution from alanine (A) to lysine (K) were 44-fold more resistant to DCV inhibition than the wild-type in vitro. A previous Brazilian research study from Malta et al. (2017) [9] identified the presence of substitution A30K in 16.1% (5/31) of monoinfected patients; however, its clinical impact in the outcome of treatment was not described. In addition, analyses of subsequent samples of patient 1 have demonstrated the persistence of mutation at residue 30 in NS5A gene for two years after the end of therapy with DCV/SOF. Other studies have also reported the persistence of NS5A RASs after treatment cessation [12,20]. These reports suggested that the occurrence of some NS5A substitutions might have conferred an evolutionary advantage for resistant variants since they are not affected by NS5A inhibitors and, even in the absence of the selective pressure imposed by antiviral drugs, these substitutions persist and do not affect viral replication level, as evidenced for viral strain studied in case report 1. This might be a matter of concern since recent publications indicate that newly approved DAAs elbasvir, ledipasvir, pibrentasvir and velpatasvir might have limited efficacy when HCV infecting strain presents substitution A30K in NS5A protein [7,21,22,23]. In conclusion, case report 1 has shown a particular case in which a molecular viral factor (RAS A30K) in combination with other factors, such as HCV subtype 3a infection and cirrhosis, might have contributed to non-response and might limit the efficacy of retreatment with other NS5A inhibitors.

In case report 2, side effects had limited the therapy conclusion with DAA telaprevir. Until now, no association of MD or past hepatitis B infection and alteration in response to treatment was reported. In addition to treatment cessation, the high viral load (6.26 Log IU/mL) could also have influenced the negative outcome. Along with high HCV RNA levels, non-response condition was corroborated by augmented liver biochemical markers, indicating active hepatic damage. Although encouraging results were seen in overcoming viral infection with a 24-week rescue therapy combining DAAs targeting different HCV proteins, SVR12 was not achieved by patient 2. Based on our resistance analyses findings, the selection of viral strains with amino acid substitution in residue 93 of NS5A protein from tyrosine (Y) to asparagine (N) could have represented a major factor associated to therapeutic failure in patient 2. Considering in vitro experiments from Wyles et al. (2017) [12], RAS Y93N could lead to a 10,000-fold reduction in susceptibility to DCV. In a previous study, our group reported the emergence of this mutation in a non-responder patient monoinfected with subtype 1a whose sample was collected after the end of therapy, suggesting that emergence of RAS Y93N after DCV-selective pressure in combination with host and viral negative predictive factors contributed to treatment failure, as well as observed here with patient 2 [10]. In summary, despite hepatitis C treatment with DAAs being considered well-tolerated and efficient in Brazil, our case report 2 suggested that identification of mutations in HCV genome, especially in NS5A gene, should be considered in particular cases when SVR rates were not achieved even with favorable factors, such as longer duration of treatment, non-cirrhotic hepatic condition and non-previous therapy with same DAA class. 

Here, two cases of DAA-failure in which the presence of RASs A30K and Y93N in HCV NS5A protein might have been an important factor for patients’ non-response were presented. Unfortunately, we were not able to perform pretreatment resistance tests since, in Brazil, physicians only request resistance analysis after DAA-therapy failure. Due to few reports to date, the clinical impact of the association between the presence of NS5A RASs A30K and Y93N in vivo and DAA-therapy non-response remains to be clarified. Nonetheless, here we report two cases of treatment failure where identifying the presence and persistence of mutant strains with NS5A RASs represented a crucial background information to guide physicians’ decision to wait for the availability of upcoming DAAs in Brazil before prescribing a rescue therapy in order to prevent the occurrence of cross-resistance to the NS5A inhibitors currently available.

## 5. Conclusions

The availability of all-oral DAA-combined therapies in Brazilian clinical guidelines for the treatment of hepatitis C represented a breakthrough in the clearance of viral infection and achievement of SVR. In counterpoint, there are still cases of treatment failure that reinforce the evaluation of viral resistance to support physicians’ conduct in a rescue therapy with different DAAs. In this report we highlighted that a combination of viral and host factors might represent negative predictive determinants in hepatitis C DAA treatment outcomes. Non-responders should be carefully evaluated, including considering resistance associated-substitution analysis, before retreatment.
